# Effect of vibration during visual-inertial integration on human heading perception during eccentric gaze

**DOI:** 10.1371/journal.pone.0199097

**Published:** 2018-06-14

**Authors:** Raul Rodriguez, Benjamin Thomas Crane

**Affiliations:** 1 Department of Bioengineering, University of Rochester, Rochester, NY, United States of America; 2 Department of Otolaryngology, University of Rochester, Rochester, NY, United States of America; 3 Department of Neuroscience, University of Rochester, Rochester, NY, United States of America; Ludwig-Maximilians-Universitat Munchen, GERMANY

## Abstract

Heading direction is determined from visual and inertial cues. Visual headings use retinal coordinates while inertial headings use body coordinates. Thus during eccentric gaze the same heading may be perceived differently by visual and inertial modalities. Stimulus weights depend on the relative reliability of these stimuli, but previous work suggests that the inertial heading may be given more weight than predicted. These experiments only varied the visual stimulus reliability, and it is unclear what occurs with variation in inertial reliability. Five human subjects completed a heading discrimination task using 2s of translation with a peak velocity of 16cm/s. Eye position was ±25° left/right with visual, inertial, or combined motion. The visual motion coherence was 50%. Inertial stimuli included 6 Hz vertical vibration with 0, 0.10, 0.15, or 0.20cm amplitude. Subjects reported perceived heading relative to the midline. With an inertial heading, perception was biased 3.6° towards the gaze direction. Visual headings biased perception 9.6° opposite gaze. The inertial threshold without vibration was 4.8° which increased significantly to 8.8° with vibration but the amplitude of vibration did not influence reliability. With visual-inertial headings, empirical stimulus weights were calculated from the bias and compared with the optimal weight calculated from the threshold. In 2 subjects empirical weights were near optimal while in the remaining 3 subjects the inertial stimuli were weighted greater than optimal predictions. On average the inertial stimulus was weighted greater than predicted. These results indicate multisensory integration may not be a function of stimulus reliability when inertial stimulus reliability is varied.

## Introduction

Determining how we are moving relative to the outside world requires processing of multiple sensory stimuli. For heading perception two salient sensory systems are visual and vestibular. Vision tells us how the world is moving relative to us using cues such as optic flow[1, 2], while the vestibular system is a more direct measure of self-motion. In addition to the vestibular system, proprioception likely plays a minor role in sensing inertial motion[3], but in this paper we will use the term inertial to include combined vestibular and proprioceptive cues. Heading can be determined from inertial motion alone[4–7]. However, when visual cues and inertial cues are available they are integrated to form a unified perception of heading direction[8–14].

Multisensory integration has previously been studied for several types of sensory modalities. Previous work on visual-inertial heading integration has found reliability and perceived direction of the combined stimulus can be estimated from the relative reliability of the individual stimuli such that each sensory cue is weighted by the inverse of its variability[9, 13–17]. However, in other sensory systems there have been significant deviations from ideal behavior[18–22]. Such deviations have also occurred in visual-inertial integration with a tendency, at least in a subset of individuals, to weigh the inertial stimulus more heavily than predicted based on its reliability[15, 23]. In their monkeys, Fetsch et al. proposed the heavier weighting of the inertial stimulus relative to optimal predictions may be due to the animals being trained on the inertial stimulus first. However, the same effect was also seen in at least some human participants who did not receive training[15]. In a recent study in which the results were reported for individual human subjects, the average performance indicated heavier weighting of the inertial stimulus relative to optimal predictions but there was significant variation between subjects[16].

One issue potentially complicating visual-inertial heading integration is the disparity in coordinate systems between the two, with inertial headings being in body coordinates and visual headings being in retinal coordinates[11, 16]. Recordings in the ventral-intraparietal area (VIP) and dorsal medial superior temporal area (MSTd) suggest visual signals in an eye centered or retinotopic reference frame [24–29] while vestibular/inertial signals are corrected to body coordinates with head rotation[8, 30]. The reference frame differences seen in MSTd and VIP persist to the level of perception[11, 16]. This presents a paradox in integration of visual-inertial stimuli because during eccentric gaze visual headings are biased by their retina coordinate system and Bayesian ideal performance predicts integration based only on reliability without considering this bias. Weighting the inertial stimulus more heavily than the Bayesian ideal may be a method of minimizing this loss of accuracy with gaze shifts. One possible explanation why prior studies have found inertial stimuli to be weighted more than expected[9, 15, 16, 23, 24, 31] is this may be a mechanism for minimizing potential errors caused by visual headings being in retinal coordinates. The current study uses eccentric gaze positions to dissociate the coordinate systems of the visual and inertial headings.

An alternative explanation for why an inertial heading stimulus may be more heavily weighted is that in prior studies only the reliability of the visual stimulus was varied while the inertial stimulus was not modified[9, 12, 15, 16, 24]. Since the reliability of the inertial stimulus remained constant subjects may have perceived it to be more reliable. Methods of altering the reliability of a visual stimulus using variable coherence are well established. Although the duration of an inertial stimulus can influence its reliability[31], a study of integration with vision requires that these stimuli be the same duration and there are no established methods for altering the reliability of an inertial stimulus while keeping the duration constant. The current study tests the hypothesis that decreasing the reliability of the inertial stimulus will decrease its relative weight during multisensory heading integration. This is done using a visual-inertial heading task during eccentric gaze positions using variable amplitude vibration in the inertial stimulus to modify its relative reliability.

## Methods

### Ethics statement

The research was conducted according to the principles expressed in the Declaration of Helsinki. Written informed consent was obtained from all participants. The protocol and written consent form were approved by the University of Rochester research science review board prior to the study being conducted.

### Human subjects

A total of 5 human subjects (2 male) completed the experiment. These were the subset of the 7 subjects who competed our previous experiment, where the reliability of the visual stimulus was modified[16], who were available to do the current experiment. The two remaining subjects were not tested because they were no longer in the area. These participants were naïve to the purpose and design of the experiment. Subjects had normal or corrected to normal vision. Ages ranged from 23–68 with a mean of 36 and standard deviation of 18. All subjects had no history of vestibular disease.

### Equipment

Inertial stimuli were delivered using a 6-degree-of-freedom motion platform (Moog, East Aurora, NY, model 6DOF2000E), similar to that used in other laboratories for human motion perception studies [15, 32–34] and previously described in the current laboratory for heading estimation studies[7, 16, 35], with the head fixed using a helmet. Binocular eye position was monitored and recorded at 60 Hz using an infrared head mounted video eye tracking system (ETD-300HD, IScan Inc, Woburn, MA), as previously described, which studied the effects of gaze direction on heading perception[16]. At the start of each session, the eye tracker was calibrated using fixation points that were at 10° up, down, right, left, and one at center. During the experiment, eye position had to be within 8° of the intended fixation point prior to delivering the stimulus. These fixation windows were purposely wider than necessary because no fixation point was visible during the stimulus (doing so tempts subjects to report the location of the focus of expansion relative to the fixation point). There were moving visual stimuli, and the subject moved in trials with an inertial component. Thus there was a potential for optokinetic nystagmus and the linear vestibulo-ocular reflex[36] to cause eye movements. Eye position was monitored in real time as well as recorded for later analysis. If both eyes strayed more than 8° in the horizontal direction from the intended position after the fixation point was extinguished and during the stimulus presentation a characteristic tone was played after the response was recorded. This alerted the technician running the experiment that the subject made a fixation error. If these errors were frequent the subject was given further instructions or the eye tracking system adjusted as appropriate. The technician running the experiment could also see the video from the eye movement tracker in real time which allowed factors such as slip of the video goggles to be identified. The post-trial analysis identified trials in which the gaze did not remain within the intended region, as with our prior study[16] fixation errors were rare and analysis used all trials because removal of the failures had minimal influence. In the current study a central gaze condition was not tested. This was because lateral gaze positions were found to have similar thresholds and reliability to the central condition in the prior study and eliminating the central condition decreased the trial burden on human subjects[16].

During all test conditions audible white noise was played over two platform-mounted speakers on either side of the subject as previously described [37]. Responses were reported using a three-button control box, the center button was used for subjects to indicate they were ready for the next stimulus. The buttons on the left and right side were used to report the direction of perceived motion after stimulus completion.

### Stimuli

**Fig 1:**
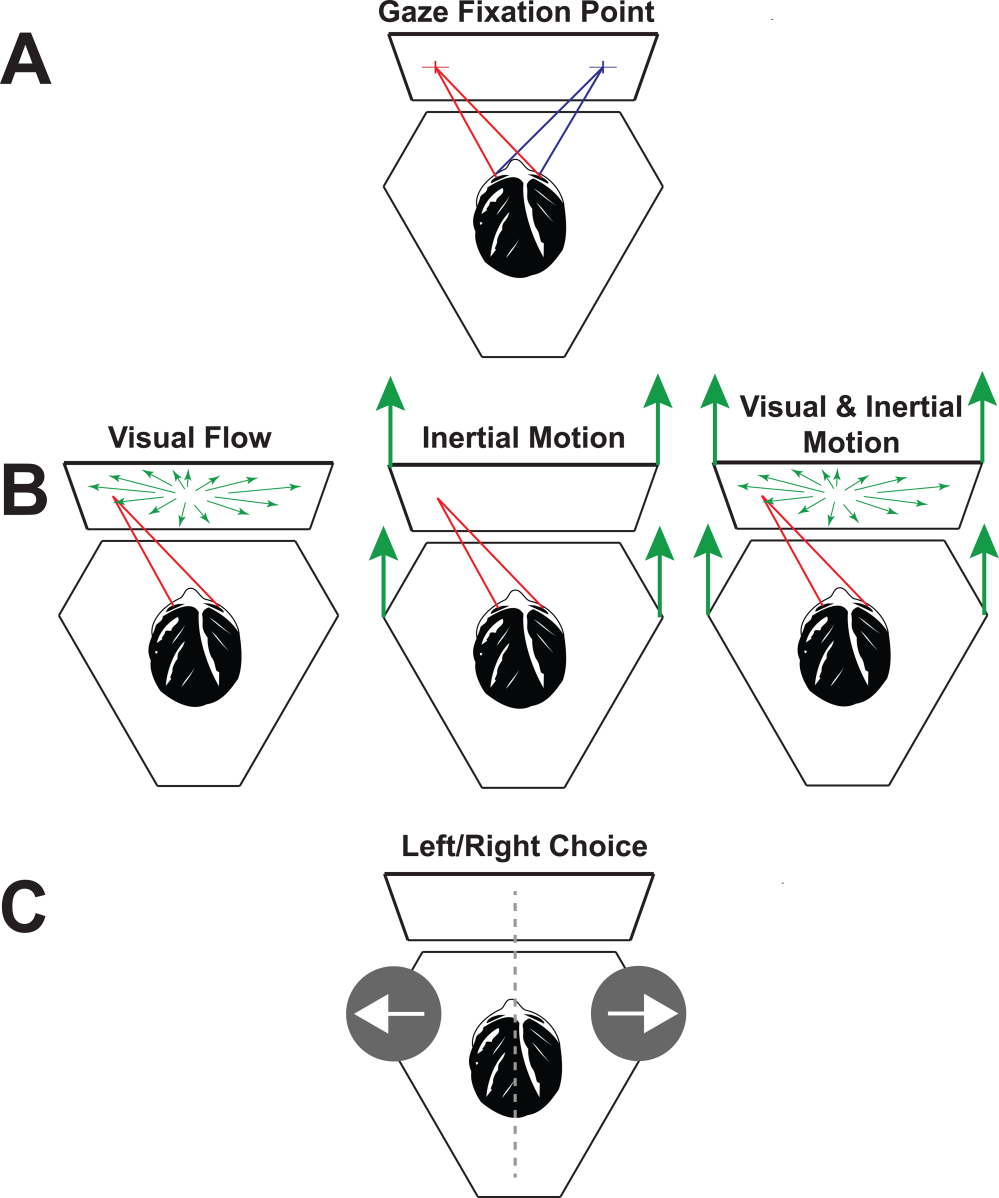
Experimental setup. The subject was seated on the motion platform in front of a display screen that was also attached to the platform. (A) Fixation point appeared at eye level that was randomly interleaved 25° to the right or left. After maintaining fixation, the subject pushed a button which extinguished the fixation point and delivered the stimulus. (B) The subject maintained gaze in the same location after the fixation point was extinguished. The stimulus could be visual, inertial (potentially with vertical vibration), or both. When presented simultaneously, the visual and inertial stimuli were always consistent with motion in the same direction. (C) After the stimulus completed there was a brief audible tone which indicated to the subject that the perceived direction of travel relative to the midline should be reported.

There were three stimulus types: visual, inertial, and combined (Fig 1). During the combined stimulus condition the visual and inertial motion were always synchronous and represented the same direction and magnitude of motion. The visual stimulus consisted of a single cycle 2s (0.5 Hz) sine wave in acceleration. The portion of the inertial stimulus in the horizontal plane also had this motion profile. This motion profile has previously been used for threshold determination[33, 37, 38] and for heading estimation[7, 35]. The displacement of both visual and inertial stimuli were 16 cm with a peak velocity of 16 cm/s and peak acceleration of 25 cm/s/s. Although the stimulus was always in a straight line it could have a left or right component with a maximum angle, relative to straight ahead, of 50° to the right or left.

The visual stimuli were presented on a 1920 x 1080 pixel color LCD screen filling 98° of the horizontal field of view that was 50 cm from the subject as previously described[16]. A fixation point consisted of a 2x2 cm cross at eye level presented 25° left or right prior to each stimulus presentation and extinguished during the stimulus presentation. The binocular visual stimulus without disparity consisted of a star field which simulated movement of the observer through a random-dot field with a star density of 0.01 per cubic cm. Each star consisted of a triangle 0.5 cm in height and width at the plane of the screen adjusted appropriately for distance. The field depth was 130 cm (the nearest stars were 20 cm and the furthest 150 cm). The visual stimulus was always presented at 50% coherence, thus during every 60 Hz visual frame data half the points were randomly repositioned. This was done to decrease the reliability of the visual stimulus so it would be similar to the inertial stimulus.

**Fig 2:**
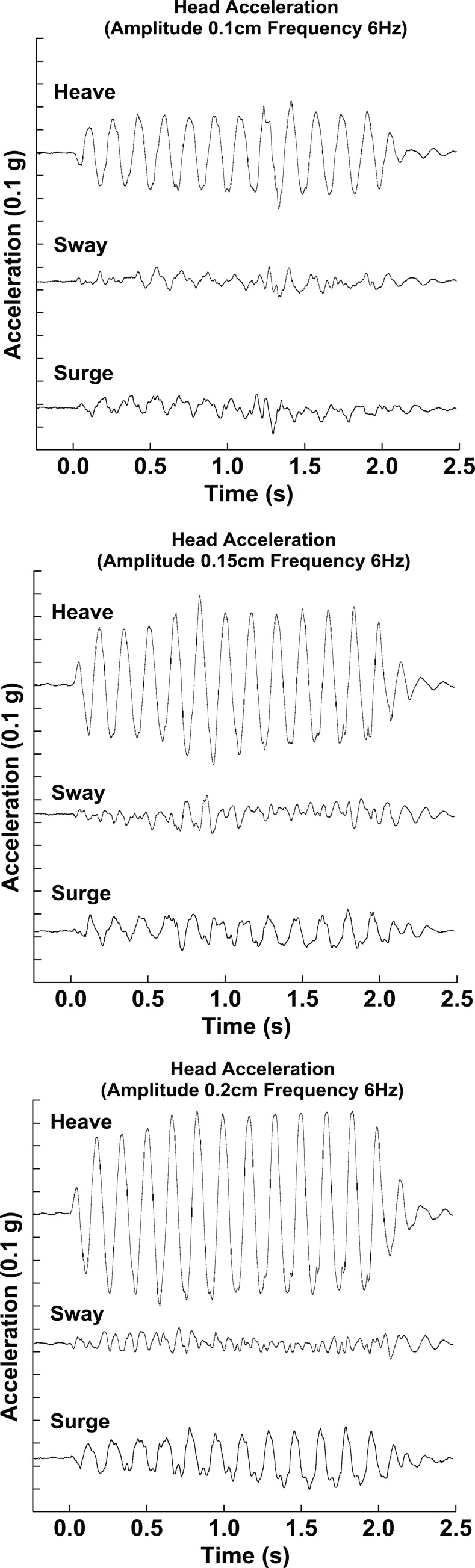
The vibration stimulus as measured from a bite bar based accelerometer. The vibration is shown for the three amplitudes of vibration tested in the current experiments– 0.10 cm (top), 0.15 cm (middle), and 0.20 cm (bottom). The primary stimulus was in the heave (vertical) direction, but there was a small component in the sway (lateral) and surge (fore-aft) directions.

Some inertial stimuli also had a vibration component delivered in the vertical or heave direction. The heave axis was chosen because it would likely have the same influence independent of heading direction, while vibration in the horizontal plane would not. This vertical vibration component varied in amplitude and was 0.00, 0.10, 0.15, or 0.20 cm at 6Hz. For the 0.20 cm amplitude vibration the peak velocity was 3.77 cm/s and peak acceleration was 0.145 g. These stimuli were verified using an accelerometer mounted to a bite bar (Fig 2).

Eye position was maintained at 25° to the right or left during trials using the method previously described[16]. Briefly, a 2x2 cm cross appeared at the desired gaze position prior to the stimulus presentation. After the subject fixated the target they pressed a button to indicate they were ready, then the fixation point was extinguished and the stimulus delivered. The fixation point was turned off to eliminate the tendency to report the visual focus of expansion relative to the fixation point. The left and right gaze positions were randomly interleaved between stimulus presentations.

### Experimental procedure

Each subject completed 4 inertial stimulus only trial blocks: Vertical vibration in each of three amplitudes and a trial block with no vibration. An additional 4 trial blocks included a 50% coherence visual motion which matched the platform motion so that it was consistent with moving through a fixed environment. Subjects understood that in multisensory conditions the visual and inertial motion were always in the same direction. A final trial block included only the visual motion with the platform stationary. Thus, the complete experiment included 9 trial blocks which were tested in an order randomized for each subject.

**Fig 3:**
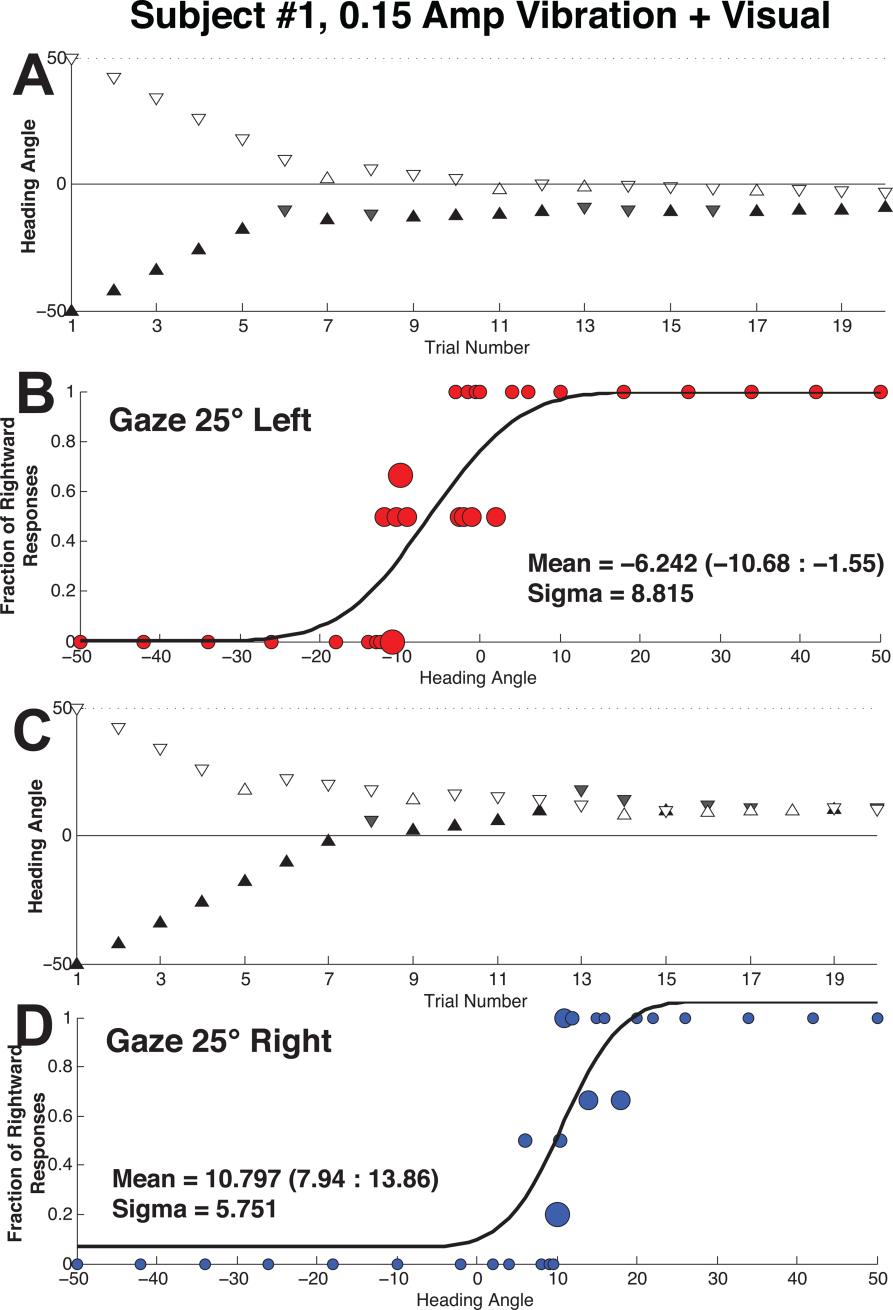
Sample data from subject #1 during the combined visual-inertial heading discrimination task. The inertial stimulus included 0.15 cm vibration and visual heading was at 50% coherence. (A) The staircase with 25° leftward gaze. (B) Represents these same responses fit by a psychometric function. Subject’s responses are represented by circles scaled proportional to the number of stimulus presentations given at that heading with the largest circles representing 5 stimulus presentations and the smallest representing a single stimulus presentation. The curve represents the best fit to the data. The mean is shown with 95% confidence intervals. (C) The staircase with 25° rightward gaze. (D) Psychometric function fit to the responses in (C).

Each trial block included 4 independent, randomly interleaved staircases: There were two staircases for each gaze direction (25° left and right). Within each gaze direction there was one staircase that started with a heading of the maximum displacement in each direction (50° left or right). All headings were defined in earth/body coordinates (the body did not rotate relative to the earth), so no coordinate transformations were needed. For all subjects the direction of the furthest displaced stimulus was unambiguous and could be reliably identified. Each staircase included 20 stimulus presentations such that the trial block included 80 stimulus presentations in a 1-up-1-down staircase with variable step size (Fig 3). Similar to the previous experiment where visual coherence was varied[16], the current experiment used an initial 8° step size which was decreased by half when the responses changed direction down to a minimum of 0.5°. Three responses in the same direction doubled the step size up to the maximum of 8°. Each staircase could pass through zero so that either staircase could deliver stimuli in either direction. This tended to focus stimuli near the point of subjective equality (PSE), or the mean of the psychometric function, at which subjects were equally likely to perceive a stimulus in either direction.

### Data analysis

The fraction of rightward responses was fit using a cumulative Gaussian function with each data point weighted based on the number of responses, at that heading, using a Monte Carlo maximum-likelihood method. After the initial data were fit, the reliability and confidence intervals were determined by randomly resampling each trial block with replacement (i.e. the same responses could be used again). This resampled data set was then curve fit to generate an estimate of the mean and sigma. The resampling and fitting was performed 2000 times, and these fits were used to determine 95% confidence intervals [16, 39, 40]. Psychometric fitting for an example trial is shown in Fig 3.

To assess the integration of visual and inertial cues, an overall visual inertial heading signal $S_{VI}$ was calculated as the weighted sum of the inertial and visual signals $S_{I}$ and $S_{V}$ (where$w_{V}=1-w_{I}$) in the form previous proposed for multisensory integration[41] with the assumption that the Bayesian prior is uniform which is appropriate for the current experiments. The assumption of uniform Bayesian priors has also been argued by others[42–44]. These equations have also been used in this form by others for heading integration tasks[17, 31, 45] and used in the current laboratory[16].

$$S_{comb}=S_{VI}=w_{I}S_{I}+w_{V}S_{V}$$ (1)

The optimal weights were calculated based on stimulus reliability and the assumption of a maximum-likelihood estimation[46]:

$$w_{V-opt}=\frac{1/\sigma_{V}^{2}}{1/\sigma_{I}^{2}+1/\sigma_{V}^{2}};w_{I-opt}=\frac{1/\sigma_{I}^{2}}{1/\sigma_{I}^{2}+1/\sigma_{V}^{2}}$$ (2)

Empirical weights were determined from the means (i.e. biases or PSEs) of the visual and inertial stimuli $\mu_{V}$ and $\mu_{I}$ given and the mean of the combined stimulus$\mu_{VI}$.

$$w_{V-emp}=\frac{\mu_{VI}-\mu_{I}}{\mu_{V}-\mu_{I}};w_{I-emp}=\frac{\mu_{VI}-\mu_{V}}{\mu_{I}-\mu_{V}}$$ (3)

Both the optimal and empirical weights were calculated for each subject for each visual-inertial condition. Ideal weights were based on the sigma of the visual condition alone ($\sigma_{V}$) and the inertial sigma ($\sigma_{I}$) calculated from the inertial condition and each of the 3 vibration amplitudes tested. Empirical weights were calculated using the mean for the visual condition ($\mu_{V}$) as reported in our prior study[16]. For consistency with the prior study, means from the 100% visual coherence condition were used to calculate empirical weights, although changing coherence had no significant effect on bias. The inertial mean ($\mu_{I}$) was determined using the same amplitude of vibration as used in the combined visual-inertial ($\mu_{VI}$) condition. Empirical weights could be calculated because gaze tended to offset perception of visual headings but had minimal influence on inertial headings. Weight calculations were also performed 2,000 times using the estimates obtained from the Monte Carlo resampling allowing 95% confidence intervals to be determined on the empirical and ideal weights.

After modeling, the fit parameters were analyzed using repeated measures analysis of variance (ANOVA). Analysis was performed using GraphPad Prism software. Differences were considered statistically significant if p < 0.05. No correction was made for multiple comparisons because differences were generally either not significant (e.g. p > 0.1) or highly significant (e.g. p < 0.001).

## Results

**Fig 4:**
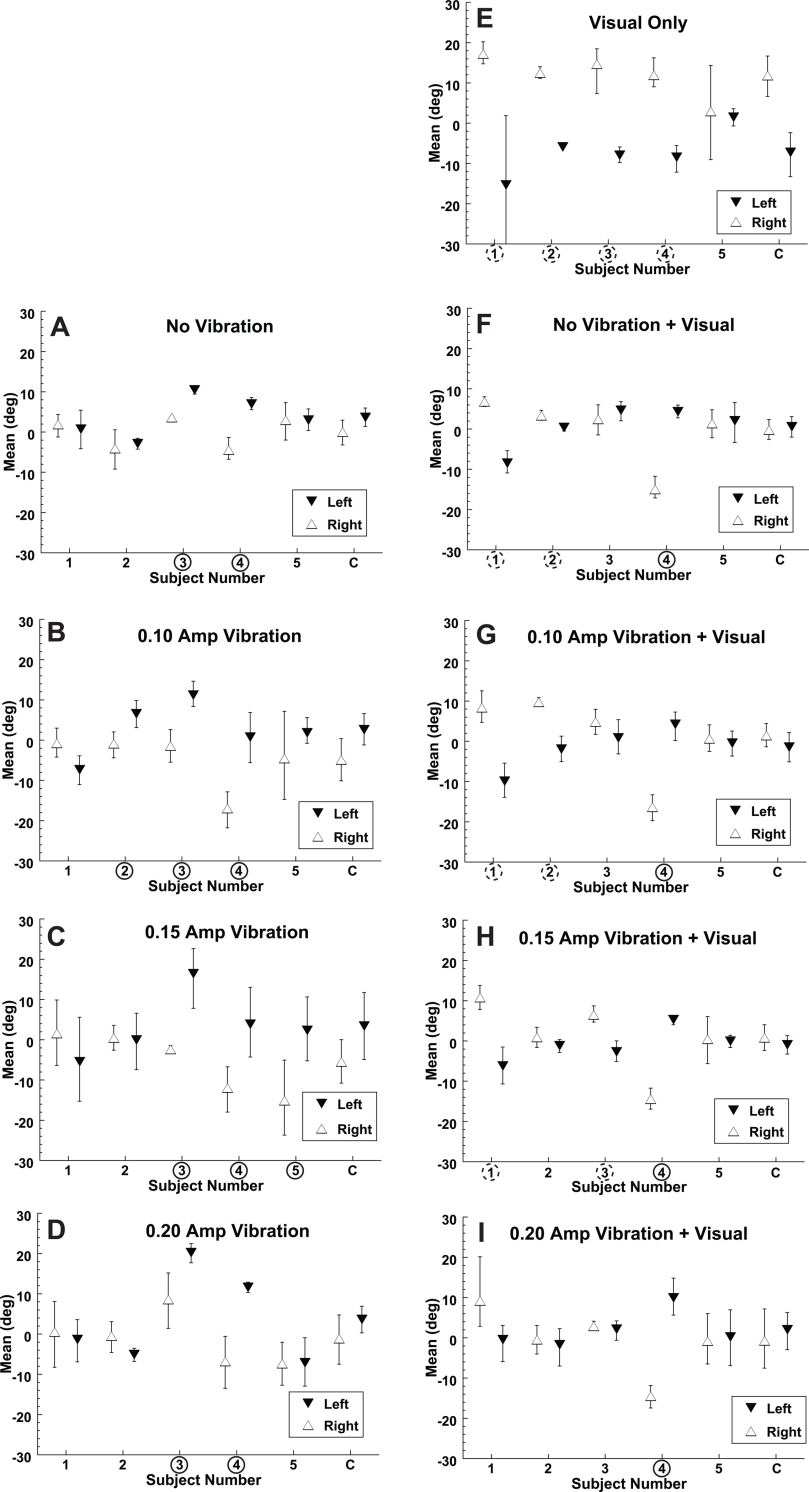
The mean or point of subjective equality (PSE) of visual and inertial heading discrimination. Each condition was tested with the eye position 25° to the left (filled downward pointing triangles) and 25° to the right (open upward pointing triangles). Error bars represent the 95% confidence interval (CI) as measured using the Monte Carlo technique. A data point shifted in the positive direction (upward) indicates that a stimulus in the midline is more likely to be perceived to the left. Subject numbers marked with a solid circle indicate the leftward gaze shifted the PSE significantly (p < 0.01 using the Monte Carlo technique) to the left when right and left gaze were compared. Similarly, a dashed circle indicates a significant effect in the opposite direction. The data combined across subjects is marked with a C in the furthest right column. (A) Indicates a purely inertial stimulus. (B-D) Inertial plus vibration is shown. (E) The visual only stimulus. (F-I) Are the multisensory, visual-inertial conditions.

Eye position had only a small effect on the bias during the purely inertial stimulus. With no vibration and left gaze the average point of subjective equality (PSE) was 3.6 ± 5.2° (mean ± SD) to the right and with right gaze the mean bias was 0.0 ± 3.9° (Fig 4A). Using a paired T-test this difference in gaze offset was not significant (p > 0.1) for the no vibration inertial motion. However small differences in bias were also present with gaze direction when vibration was added to the inertial stimulus. When all inertial conditions were considered across vibration amplitudes (Fig 4B–4C) the PSE was shifted 3.3 ± 7.7° (mean ± SD) to the right with left gaze and 2.9 ± 6.3° to the left with right gaze. These were significantly different (p = 0.006, paired T-test). Thus, a midline inertial stimulus would be more likely to be perceived in the direction of gaze. With a visual stimulus the bias with gaze shifts was larger than seen with the inertial stimulus and in the opposite direction (Fig 4E), such that the mean PSE was 11.8 ± 5.4° to the right with right gaze and 7.3 ± 6.0° to the left with left gaze. Thus, for the visual condition there was a significant difference in PSE based on gaze direction (p = 0.02, paired T-test). Because of this shift a midline visual heading would be likely to be perceived opposite the gaze direction. Thus, gaze shifts had an opposite effect on the direction of visual and inertial heading perception such that at lateral gaze positions the difference between the perceived direction of visual and inertial headings (when delivered separately) averaged 11.4°. This offset allowed the relative weights of visual and inertial headings to be determined during a multisensory stimulus presentation (empirical weight, Eq 3).

**Fig 5:**
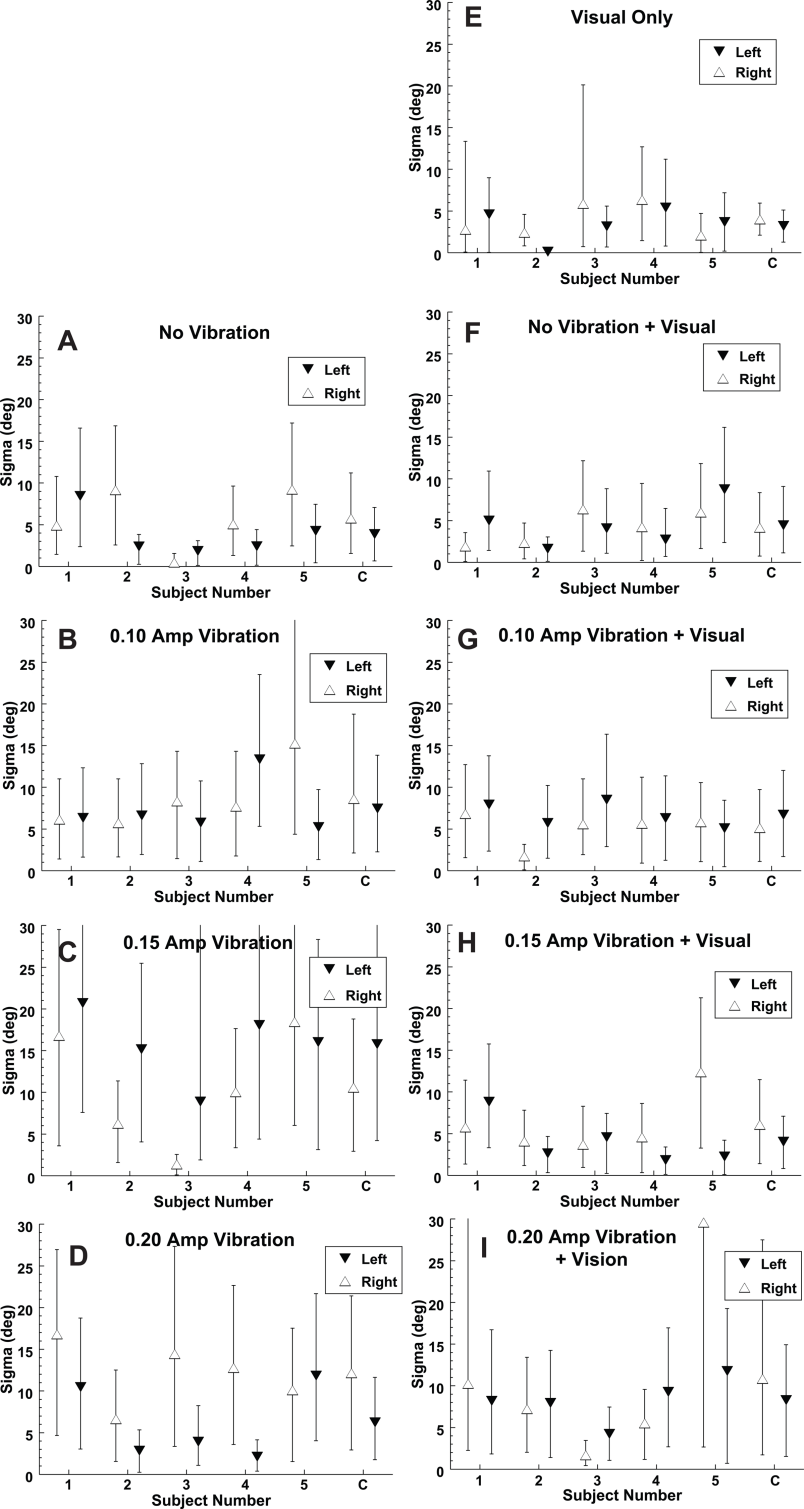
The thresholds of visual and inertial heading discrimination. As with Fig 4 the symbols represent left and right gaze positions. Error bars represent the 95% CI for each subject (numbered) and the combined data (C). (A-D) Purely inertial stimulus while on the right (E-I) include a visual component.

The reliability of the visual and inertial headings was measured using the slope of the psychometric function or sigma (Fig 5). The average sigma for the 50% visual coherence heading was 3.6 ± 2.0° and for the inertial heading without vibration it was 4.8 ± 3.2°. In subjects 3 and 4 the reported inertial heading was more reliable (i.e. smaller sigma) and in the other 3 subjects (1,2 and 5) the reported visual heading was more reliable and overall within the group there was no significant difference between them (paired T-Test, p = 0.3). This was by design as the 50% visual coherence visual stimulus was chosen to make the visual and inertial stimuli near equal in reliability so that multisensory integration could be effectively studied.

**Fig 6:**
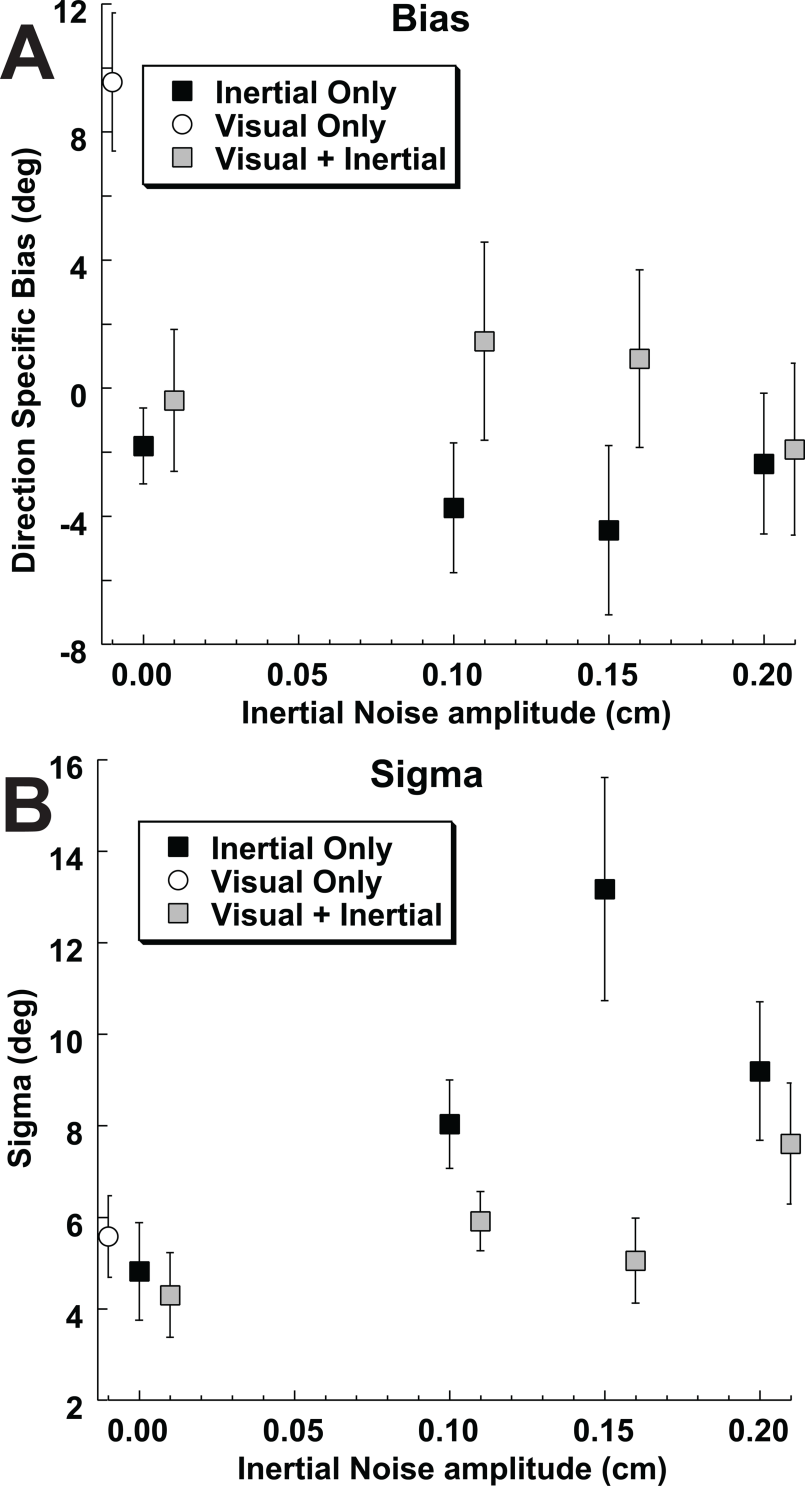
Bias and thresholds of visual and inertial headings across subjects and gaze directions. The bias of the left gaze was negated so the average bias relative to eye position could be considered (e.g. the mean with the left inverted). Error bars represent ±1 SEM. Conditions with the same amount of vibration were offset slightly to keep the data points from overlapping. The purely visual condition is shown with an open circle. Inertial only with a filled square. The combined condition is shown by a gray square. (A) Bias or PSE. (B) Threshold or sigma.

When vertical vibration was added to the inertial heading stimulus, it significantly increased the sigma (i.e. decreased the reliability) relative to the no vibration stimulus (p < 0.001, paired T-test). Sigma averaged across left and right gaze positions for the inertial stimulus was 4.8 ± 3.2° with no vibration and 8.8 ± 2.6° for all amplitudes of vibration. Any effect of the amplitude of vibration on the reliability of the inertial stimulus was less clear. Trials with vibration had a larger sigma than those without, but in trials with vibration the vibration amplitude was not associated with the sigma (ANOVA, p > 0.1; Fig 6B).

**Fig 7:**
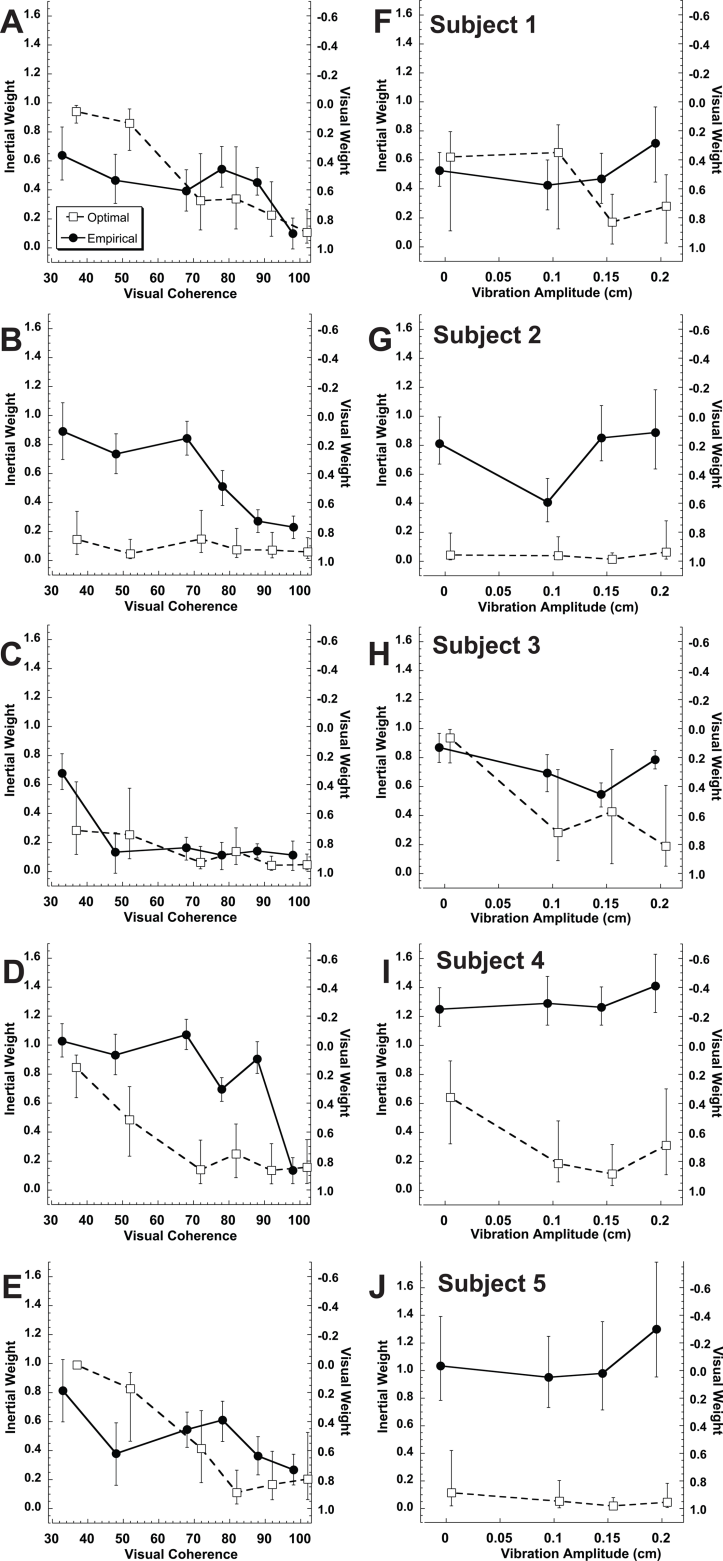
The relative weights of the visual and inertial stimuli determined for each subject based on the relative reliabilities of the stimuli. The sum of the visual and inertial weights is always one. Weights determined empirically from bias are shown as filled circles and solid lines. Optimal weights were determined from average thresholds and are represented by open squares and dashed lines. Error bars represent the 5^th^ and 95^th^ percentile for the weights determined from 2,000 random resamplings for each condition. The empirical and optimal weights were artificially horizontally offset from each other for clarity. The left side of the figure (A-E) represents the subjects in this study when the visual coherence was varied in the prior study[16]. The right side of the figure (F-J) represents the current experiments which used vibration to vary the reliability of the inertial stimulus.

**Fig 8:**
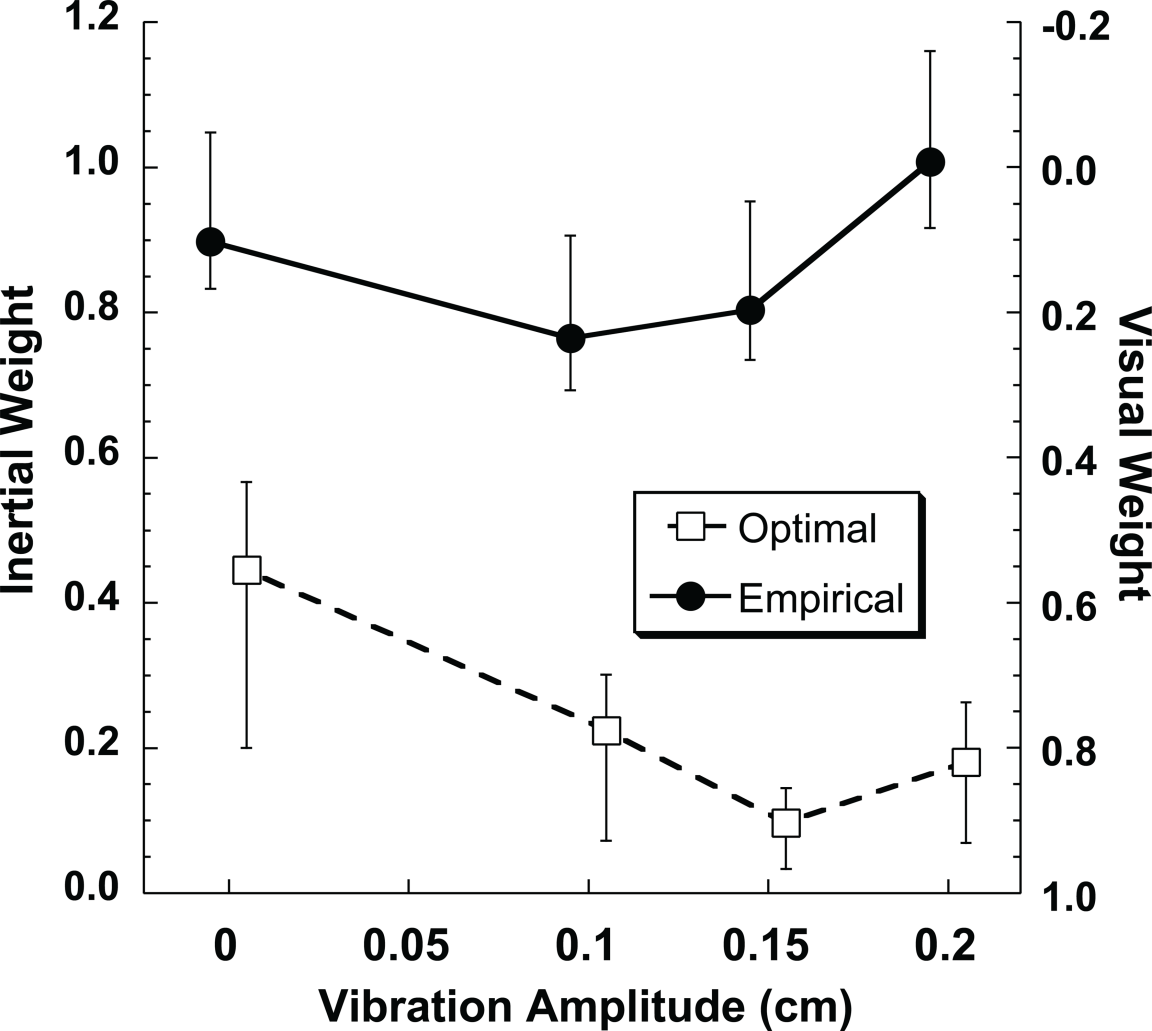
The relative weights of the inertial and visual stimuli averaged across the five subjects. Empirically calculated weights based on bias are shown as filled circles with a solid line. Optimally calculated weights based on thresholds (e.g. stimulus reliability) are shown as open squares with a dashed line. As with the individual data the error bars represent the 5^th^ and 95^th^ percentile determined from 2,000 random resamplings for each condition.

Although there tended to be a large bias in heading perception in the opposite direction from gaze in the visual only condition, this bias largely disappeared when visual and inertial stimuli were combined (Fig 6A). The relative weights of the visual and inertial stimuli for the combined heading condition were calculated. Optimal weights were determined using the relative reliabilities (sigma) measured in the single modality conditions. Empirical weights were determined by comparing the mean (bias) of the multimodal condition with that of the single modality visual and vestibular condition. These had previously been measured in these same subjects while varying visual coherence[16], which is shown for reference in Fig 7A–7E. In the current study these weights were determined while varying the reliability of the inertial stimulus in these same participants (Fig 7F–7J). In both studies 95% confidence intervals were measured using a Monte Carlo technique based on random resampling of the responses. In subjects 1 & 3 the empirical weights of the visual-inertial heading was near the optimal prediction in both the current study (Fig 7F and 7H) and the previous study in which the reliability of the visual stimulus was varied (Fig 7A and 7C). In subjects 2 & 4 the inertial heading was weighted more than was predicted based on its relative reliability when both the inertial reliability was varied (Fig 7G and 7I) and when the visual reliability was varied (Fig 7B and 7D). The final subject (#5) weighted the visual and inertial stimuli near equally when the visual reliability was varied (Fig 7E) but put nearly all the weight on the inertial heading when the inertial stimulus was varied (Fig 7J). On average, the empirical weight of the inertial stimulus was much higher than optimal predictions (Fig 8). Without vibration the average optimal inertial weight was 0.47 while the empirical inertial weight was 0.90. Due to the large variation between subjects these weren’t significantly different (paired T-test p = 0.11). However, when averaged across vibration conditions, the optimal inertial weight was 0.19 while the empirical weight was nearly unchanged at 0.87. This difference was highly significant (p < 0.001), demonstrating that the inertial stimulus was weighted greater than predicted based on its reliability in these experiments.

## Discussion

Several papers have now examined visual-inertial cue integration for heading estimation[9, 15, 16, 24]. On average all of these studies have found that the inertial (i.e. vestibular) cue is weighted higher than would be predicted based on its relative reliability. When the weights for individual subjects were reported there was significant variation in the relative weights of the visual and inertial stimuli in both monkeys[15] and humans[16]. In these individual weights it seemed that the higher weight of the inertial stimulus was due to behavior in one of three monkeys[15] and two of seven humans[16]. The reason the inertial stimulus is more heavily weighted than expected, has not been fully explained. In the monkey experiments it was thought that this may be due to animals being trained on the inertial cue first, but this would not explain why this trend was also present in humans[15, 16, 23, 31] who were not trained. The current experiments investigated the possibility that the higher inertial weighting was due to the inertial cue reliability being constant unlike the visual cue reliability which was varied between trials. The current results demonstrated that the inertial continued to be weighted more heavily even when the reliability was varied. Surprisingly, in subjects 3, 4, & 5 (Fig 7), with the current protocol, the inertial stimulus was consistently weighted greater than in the previous experiments where the visual stimulus reliability was varied. In some individuals (e.g. subject 4 in Fig 7) the empirical visual weight was negative (i.e. the inertial weight was greater than unity). This isn’t possible when using Bayesian predictions, and in both these subjects the inertial stimulus was weighted heavier than it was when the reliability of the visual stimulus was varied. In our analysis we assumed that subjects had no top-down expectations or biases also known as priors about the stimuli as others have[17, 31, 42–45] and we did in our previous study[16]. It is possible that subjects came into these experiments with priors that did not have a uniform influence on perception and a *P*(X) might be able to explain the current findings in terms of a Bayesian model. However, if such priors existed it is unclear what they were or how they could be described in the model. Thus, the best evidence was that multisensory integration was non-Bayesian (i.e. the weights of the visual and inertial cues were not based on their reliability) in this paradigm.

There is not a well-established method of decreasing the reliability of an inertial heading stimulus, which is likely why previous work on visual-inertial heading integration has varied the reliability of only the visual stimulus by modifying its coherence[9–11, 15, 16]. In the current experiments a vertical vibration was used and shown to decrease the reliability of heading estimation in the horizontal plane. However, this finding is likely to be surprising to some and perhaps controversial due to known effects of stochastic resonance (SR) in which increasing the noise level of the input has improved the signal-to-noise level of the output[47]. Some studies have demonstrated that low levels of vestibular stimulation improve balance function when applied as either vibration[48, 49] or galvanic stimulation[50]. In the current studies the addition of vibration decreased the reliability of the inertial headings. Thus, we did not observe an SR effect, and saw decreased reliability with addition of vibration. There are two potential reasons why the addition of vibration did not cause a SR effect. First, the vibration was perpendicular of the plane of motion, although some motion still occurred in the horizontal plane (Fig 2). It is possible that if the vibration were in another direction (e.g. sway) an effect could have been apparent. Second, the amplitude of the vibration may have been outside the appropriate range needed to induce a SR effect. Third, the frequency of vibration used may not have been appropriate for SR, others reporting the effect have used 1.1 Hz[49].

Although the addition of a vertical vibration decreased the reliability of the inertial stimulus, once the vibration was present, increasing amplitude had no clear effect on the stimulus reliability. In some preliminary experiments we tried increasing the amplitude beyond what was reported here but it became too uncomfortable for the subjects. It is also possible that amplitudes of vibration below the smallest currently tested (amplitude 0.1 cm) would have allowed some dose effect or SR. Unfortunately, it was not practical to test lower amplitudes with the current protocol because small vibrations tended to be damped out by the body, so what reached the skull was minimal and no longer in the intended direction and the resolution of the platform limited the accuracy of very small movements. Other methods may be able to investigate potential effects of lower amplitude vibration, but these were not practical to investigate as part of the current experiments. Thus, unlike varying visual coherence, using the vibration protocol described here it was not possible to have fine control over the reliability of the stimulus. Decreasing visual coherence actually decreases the amount of reliable visual information available but adding vibration did not decrease the amount of inertial information since the motion within the horizontal plane remained the same. The addition of vibration, did not decrease amount of inertial information as the motion in the horizontal plane was still the same. The amount of inertial information might be reduced by changing the amplitude of motion, but the amplitude of inertial motion could not be changed independent of the visual stimulus amplitude while keeping the two consistent with the same movement. It should also be considered that the vertical motion of the vibration was primarily a stimulus to the saccule while the horizontal motion is sensed by the utricle[51, 52]. Thus, there may be better methods of decreasing the reliability of inertial cues in the horizontal plane, but nothing has yet been established and to the knowledge of the authors there is not a method analogous to decreasing visual coherence.

Another concern about using vertical vibration in the inertial stimulus is that no such vibration was present in the visual stimulus which has the potential to cause the stimuli to be dissociated. There is no evidence that this occurred. The bias in the no vibration condition was similar to the vibration condition (Fig 6A). Furthermore, the sigma was decreased in the combined condition (Fig 6B) when vibration was present which would not be expected if the stimuli were perceived as independent.

These findings are most consistent with the visual and inertial cues forming a common perception of heading. Even though the visual and inertial headings were always the same, they were perceived to have different directions with eccentric gaze in the unisensory conditions. It has previously been shown that even when there are large offsets between visual and inertial headings they form a common perception[14, 17, 53]. If subjects perceived visual and inertial headings as different and chose to alternately report one sensory condition or the other in the multisensory condition, one would still expect to find a PSE intermediate between the visual and inertial unisensory conditions. Such a strategy is not consistent with the current observations because with such a strategy the PSE would not depend on the relative reliability of the sensory stimuli and the sigma in the combined condition would be larger than the difference between the means of the two unisensory conditions for parameters relevant to current experiments when the difference in means was larger than the sigmas. Furthermore, if subjects were randomly choosing between the two sensory conditions, it could be expected that the bias would be near the mean of the two conditions. However, the data demonstrate that the bias of the combined condition was almost always close to the inertial bias despite the larger sigma seen for the inertial stimulus. None of these effects were observed and these stimuli were not felt by subjects to have different directions when presented together, although we did not include judgments about this in the experimental protocol.

One potential reason inertial cues may have a greater weight in perception, than its reliability suggests, is that this may be a mechanism for compensating for retinotopic coordinates associated with visual stimuli. A retinotopic or eye-centered reference frame has been demonstrated in multiple areas of visual processing in the medial temporal area[54], posterior parietal cortex[55], and the medial superior temporal area, which is thought to have a key role in visual heading perception[8, 28, 56]. Retinal coordinates for visual stimuli continue to the level of perception with reach planning[57–59] and heading determination[11, 16]. Despite this, it is recognized that retinal coordinates are problematic for perception as gaze shifts occur independent of head and body movements which could interfere with the perceived stability of the external world[60–62]. For heading perception, errors with gaze shifts may be minimized during driving and ambulation by maintaining gaze in the direction of travel[63–65]. However, there are common situations in which it is necessary to shift the gaze away from the direction of travel. One way to minimize errors during such gaze shifts would be to put more perceptual weight on the inertial heading which are in body coordinates[8, 30]. The current data provides further support that the inertial heading is considered greater than its reliability suggests, and this could be a mechanism for correction of eye-centered coordinates.

Bayesian integration tells us that sensory cue integration occurs in a statistically optimal manner based on the relatively reliability or precision. However, Bayesian theory assumes that both sensory cues have common causality. Sensory cues can be artificially offset as a method of studying integration, but in these cases the offsets are small and thought to go unnoticed by experimental subjects[8, 15, 19–21]. However, the current study did not artificially offset sensory cues as a perceptual offset occurs with gaze shifts such that visual headings become systematically inaccurate relative to the inertial cue. Non-Bayesian sensory integration in the current experiments could be explained by the inertial cue being known to be more accurate than the visual cue during eccentric viewing due to visual cues being in retina coordinates that are offset by gaze.
